# The Effect of Biomechanical Loading Parameters on the Stress and Strain Behavior of Orthodontic Mini-Implants: A Finite Element Study

**DOI:** 10.3390/jfb17030114

**Published:** 2026-02-27

**Authors:** Tinela Panaite, Cristian Liviu Romanec, Bogdan Radu Dragomir, Ana Sîrghie, Carmen Amititeloaie, Carina Balcos, Carmen Diana Nicoleta Savin

**Affiliations:** Grigore T. Popa University of Medicine and Pharmacy Iasi, 700259 Iași, Romania; tinela-panaite@umfiasi.ro (T.P.); liviu.romanec@umfiasi.ro (C.L.R.); radu.dragomir@umfiasi.ro (B.R.D.); carmen.amititeloaie@umfiasi.ro (C.A.); carina.balcos@umfiasi.ro (C.B.); carmen.savin@umfiasi.ro (C.D.N.S.)

**Keywords:** anchorage stability, finite element analysis, insertion depth, loading direction, orthodontic force, orthodontic mini-implants, primary stability

## Abstract

Background/Objectives: This study evaluated the influence of key biomechanical parameters—orthodontic force magnitude, loading direction, and insertion depth—on stress and strain distribution in orthodontic mini-implants using three-dimensional finite element analysis (FEM). Methods: A three-dimensional model of a titanium orthodontic mini-implant inserted into a mandibular bone segment was developed and analyzed under varying force magnitudes (1–10 N), loading directions (30°, 45°, and 60°), and insertion depths (2–4 mm). Cortical and cancellous bone components were included, and static loading conditions were applied using simplified, linear elastic material assumptions. Results: Stress and strain levels increased with higher force magnitudes, with implant stresses approaching critical values at loads above 9 N. Cortical bone stresses remained within physiological limits, whereas cancellous bone exceeded the microdamage strain threshold at forces greater than 3 N. A 60° loading direction reduced implant bending and strain, while deeper insertion significantly decreased strain and displacement, indicating improved primary stability. Conclusions: Within the limits of this computational model, optimal mechanical behavior was observed under 1–3 N forces, a 60° loading direction, and a 2–4 mm insertion depth. Loads above 9 N approached fatigue and interfacial risk. These findings provide computational insight into the biomechanical behavior of orthodontic mini-implants under the modeled conditions.

## 1. Introduction

The success of orthodontic mini-implants is influenced by a complex interaction of mechanical and biological factors that affect their primary and long-term stability. Reported failure rates range from 6.9% to 28%, depending on skeletal pattern, insertion site, applied force magnitude and direction, cortical bone characteristics, and operator experience [1,2]. These variations highlight the importance of understanding the biomechanical behavior of mini-implants under different clinical conditions.

Orthodontic forces applied to mini-implants must be sufficient to achieve effective tooth movement while preserving anchorage stability. Clinically accepted force magnitudes typically range between 1 and 3 N, depending on bone quality and treatment objectives, as higher forces may increase stress concentrations and micromovements at the bone–implant interface [3,4].

Insertion angle plays a critical biomechanical role by influencing bending moments, load transfer mechanisms, and the extent of cortical bone engagement. Finite element studies have commonly investigated insertion angles of 30°, 45°, and 60°, as these represent frequently used clinical ranges. Several authors have reported increased stress concentrations at lower angulations due to larger bending moments acting at the implant neck [5,6,7], whereas more vertical orientations tend to reduce bending and enhance stability [8]. However, the reported magnitude and clinical relevance of these effects vary among studies.

In addition to insertion angle, cortical bone thickness and insertion depth are key determinants of mini-implant stability. A minimum cortical thickness of approximately 1.0 mm has been suggested for adequate primary stability, while increased cortical engagement and deeper insertion have been associated with improved anchorage and reduced failure risk [9,10,11].

Recent finite element investigations have extensively analyzed the biomechanical behavior of orthodontic mini-implants under varying clinical conditions, including force magnitude, insertion angle, cortical bone thickness, and loading direction [12,13,14,15].

Several studies have demonstrated that oblique loading increases the stress concentration at the implant neck due to amplified bending moments compared to axial loading [16,17,18]. Moreover, parametric FEM studies evaluating combinations of force magnitude and angulation have shown that increased transverse force components significantly elevate bending stresses and strain concentrations in the peri-implant region [19,20].

Therefore, a comprehensive parametric evaluation integrating orthodontic force magnitude, loading direction, and insertion depth within a unified three-dimensional finite element framework is needed to better clarify their combined influence on mini-implant biomechanics. Finite element modeling provides a powerful, non-invasive approach for systematically analyzing stress and strain distributions under controlled conditions.

Previous FEM studies have investigated force magnitude, insertion angle, or depth individually, but rarely their combined biomechanical effects in a unified 3D model [2,5,6,7,8]. Furthermore, quantitative data on fatigue and microdamage-related stress–strain thresholds remain scarce.

Accordingly, the aim of the present study is to investigate the combined effects of orthodontic force magnitude, loading direction, and insertion depth on stress and strain distribution in orthodontic mini-implants and surrounding bone using three-dimensional finite element analysis.

## 2. Materials and Methods

### 2.1. Geometric Modeling

**Figure 1:**
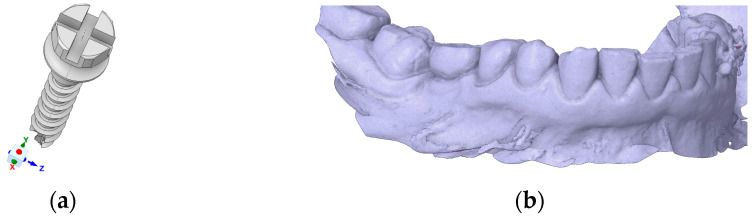
Procedure for finite element modeling of an orthodontic mini-implant with adjacent bone structures: (**a**) A commercially available titanium mini-implant (2.0 mm diameter) was reconstructed and analyzed using the finite element method; (**b**) Mandibular geometry obtained from CT scan data, exported in STL format.

A commercially available titanium mini-implant (Ti-6Al-4V) with a diameter of 2.0 mm and a length of 12 mm (Jeil Medical Corporation, Seoul, Republic of Korea) was modeled using the finite element method (FEM) (Figure 1a). The mini-implant featured a threaded portion of approximately 8 mm, allowing insertion depths ranging between 2 and 4 mm, as analyzed in the present study.

The mandibular geometry was obtained from computed tomography (CT) scans (DEXIS, Biberach, Germany) and digitized to generate a three-dimensional solid model (Figure 1b). Both the mini-implant and the mandible were reconstructed and assembled using SpaceClaim 2023.1 software (ANSYS Inc., Canonsburg, PA, USA).

### 2.2. Simulation Parameters

The insertion site of the mini-implant was selected in the interradicular space between the premolar and molar regions. The complete model was imported into ANSYS Workbench (version 2021, ANSYS Inc., Canonsburg, PA, USA) for analysis. The mechanical properties of all modeled components were defined assuming linear elastic behavior, characterized by their Young’s modulus and Poisson’s ratio (Table 1).

**Figure 2:**
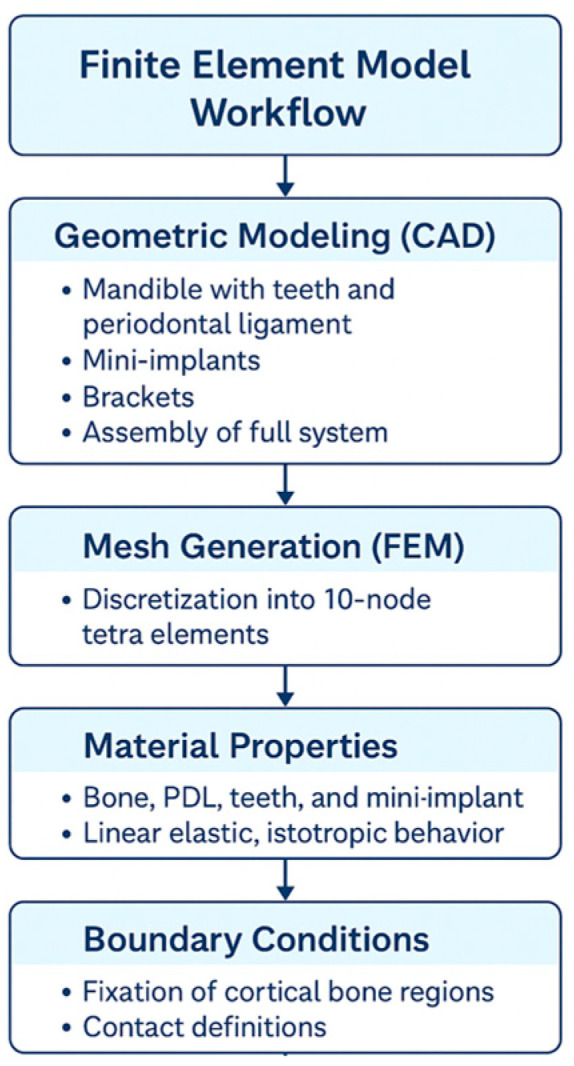
Stepwise workflow for FEM modeling of orthodontic mini-implant and mandibular bone.

The geometry was discretized using 10-node tetrahedral structural elements with automatic meshing. All components—bone, teeth, periodontal ligament, and implant—were assumed to be homogeneous, isotropic, and linearly elastic (Figure 2). These assumptions are commonly adopted in orthodontic finite element studies to reduce computational complexity and to enable controlled comparison of relative stress and strain trends across parametric conditions.

### 2.3. Boundary Conditions and Loading

The lower region of the cortical bone was fully constrained to prevent rigid body motion, simulating fixation to the remaining mandibular structure. A nonlinear contact was defined between the mini-implant threads and the surrounding bone, allowing for separation under tensile load and compression transfer through the interface. Orthodontic forces ranging from 1 N to 10 N were applied at the mini-implant head, following three different loading directions (30°, 45°, and 60° relative to the implant’s longitudinal axis) to replicate clinically relevant conditions. The loading point corresponded to the area where the orthodontic wire or spring would typically exert force in vivo.

In finite element models of orthodontic mini-implants, cortical bone thickness is a critical parameter influencing stress distribution and implant stability. For mandibular simulations, cortical bone thickness values commonly range between 2 and 3 mm, reflecting the greater cortical density and thickness of mandibular bone reported in the literature [22,23].

### 2.4. Mesh Convergence and Model Validation

A mesh convergence test was conducted to ensure numerical accuracy and independence of the results from the element size. The element size was gradually refined until the variation in maximum von Mises stress was less than 5% between successive refinements. The final model contained approximately 300,000 to 400,000 tetrahedral elements, depending on the analyzed configuration.

Model validation was achieved by comparing the predicted stress and displacement ranges with published experimental and numerical data from previous FEM studies on orthodontic mini-implants [24,25,26].

### 2.5. Analysis of the Parameters Imposed in the Finite Element Analysis

The parameters investigated in relation to the state of displacements, deformations, and stresses in the bone and implant were: force magnitude, orthodontic force angulation, insertion angle relative to the Z axis, insertion angle relative to the Y axis, mini-implant exposure length, mini-implant type, mini-implant material, and the presence or absence of the disc.

In total, 42 finite element mini-implant models were analyzed (3 orthodontic force angulations, 3 insertion angles relative to the Z axis, 3 insertion angles relative to the Y axis, 3 mini-implant exposure lengths, 10 force magnitudes, 2 mini-implant materials, 2 mini-implant types, and the presence or absence of the disc).

The orthodontic force angulation was defined as the angle between the line of the applied force and the axis parallel to the long axis of the tooth in the sagittal plane of the mini-implant head.

The insertion direction of the mini-implant was determined according to the longitudinal axis of the mini-implant in relation to the bone surface. In Table 2, the parameters providing an overview of the configuration and characteristics of the finite element model (FEA) used in this study are presented.

A nonlinear frictional contact was defined between the mini-implant threads and the surrounding bone using a Coulomb friction model with a friction coefficient of μ = 0.3. Bonded contact was applied to the remaining interfaces.

The value μ = 0.3 was selected in accordance with commonly reported values used for bone–titanium interface modeling in dental/orthodontic finite element studies [27,28].

**Figure 3:**
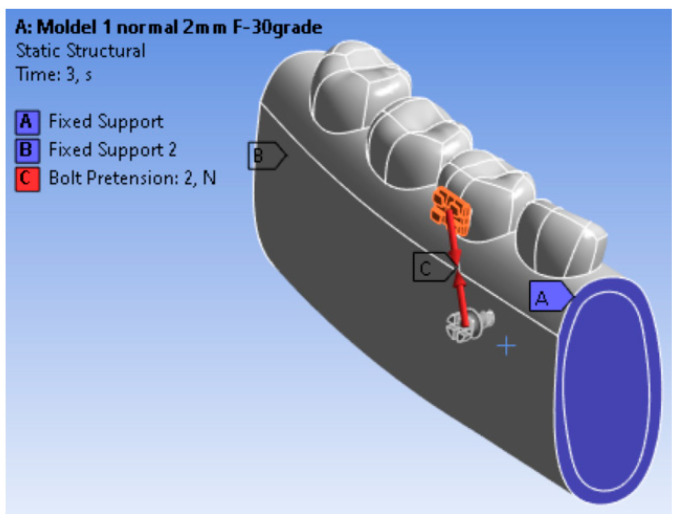
Finite element model showing the applied boundary conditions and load (red arrow indicates the direction of the applied load).

In Figure 3 the boundary conditions of the 3D geometric model and the types of contacts used in the simulation are presented.

After automatic mesh generation, the model was divided into a large number of elements and nodes, considering that the accuracy of the finite element solution increases with a higher number of elements and nodes. A finer mesh was applied in the region of mini-implant insertion, both in the bone and in the mini-implant [27]. The three-dimensional CAD model (corresponding to the geometric model) was automatically discretized into finite elements using ANSYS software.

Table 3 presents the number of elements and nodes used in the finite element analysis models.

**Figure 4:**
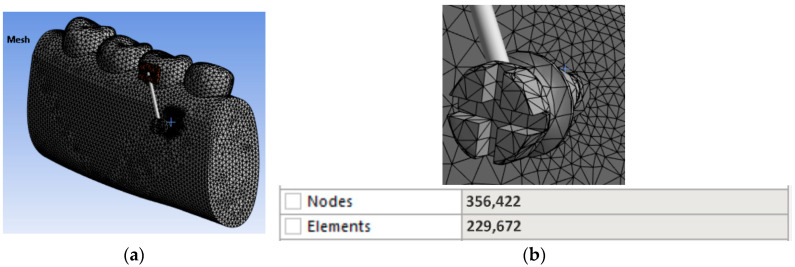
(**a**) Global 3D geometric model discretized into finite elements; (**b**) magnified discretized model in the region of interest (mini-implant and adjacent orthodontic anchorage area).

In Figure 4, the 3D geometric model discretized into 10-node tetrahedral finite elements is presented, along with the resulting number of nodes and elements.

### 2.6. Post-Processing and Evaluation Parameters

The simulations were performed using ANSYS Workbench 2021 (ANSYS Inc., USA) under static structural analysis. The results were evaluated in terms of:

- Von Mises equivalent stress (σvM) in the implant and surrounding bone;
- Total displacement (U) at the implant head;
- Contact pressure distribution at the bone–implant interface.

The obtained data were analyzed to identify areas of maximum stress concentration and to assess the influence of biomechanical parameters on implant stability and load transfer efficiency.

### 2.7. Model Verification

No direct experimental validation was performed. The present model was verified through quantitative comparison with existing FEM studies using similar geometries and force ranges. Accordingly, the results should be interpreted as relative biomechanical trends rather than absolute clinical predictions.

## 3. Results

### 3.1. Contact Pressure and Interface Behavior

**Figure 5:**
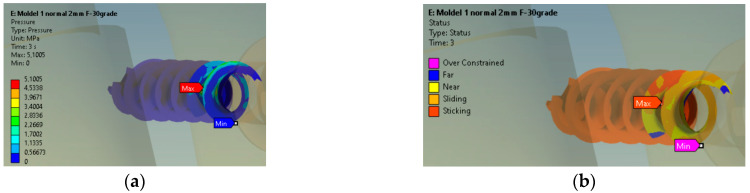
Contact pressure distribution along the implant thread: (**a**) Contact pressure distribution along the mini-implant thread (**b**) Status of linear and nonlinear contact elements.

Figure 5 illustrates the contact regions between the mini-implant and the surrounding bone. The contact pressure distribution is presented in Figure 3, showing a maximum value of 5.1 MPa. The highest contact pressure was observed near the implant neck and along the first threads.

Figure 5b presents the contact status map between the implant and cortical bone. “Sticking” regions were primarily identified along the upper and middle threads. The maximum contact pressure was localized near the implant neck, while lower contact values were observed toward the apical region. Limited “sliding” areas were detected at the interface.

### 3.2. Influence of Orthodontic Force

**Figure 6:**
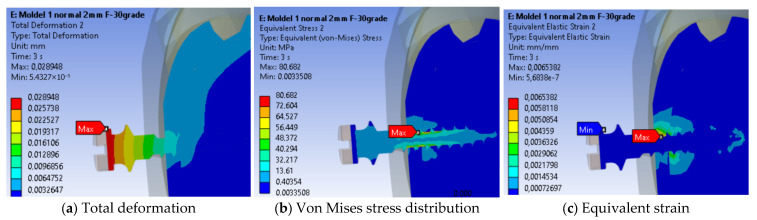
Sectional results—mini-implant.

In Figure 6a, the total displacements in the cross-section of the anchorage area—including the mini-implant, cancellous bone, and cortical bone—are shown. The maximum displacement recorded was 0.028948 mm at the head of the mini-implant near the site of orthodontic force application.

In Figure 6b, the equivalent von Mises stress distribution in section view is presented. The maximum value of 80.682 MPa was observed near the orthodontic anchorage at a depth of 1.2 mm in the cancellous bone, with the highest stress located within the mini-implant. The affected bone volume is small, and the volume of affected cortical bone is slightly larger due to its higher stiffness.

Figure 6c shows the equivalent strain distribution in the section. The maximum equivalent strain was 0.0065382 mm/mm near the orthodontic anchorage, at a depth of 0.6 mm in the cortical bone. The affected region is concentrated around the implant anchorage, with a higher proportion in the cortical bone, which has a lower elongation at the fracture than cancellous bone. However, these maximum values are localized within a very small volume of material, adjacent to the mini-implant thread.

#### 3.2.1. Influence of Force Magnitude

**Figure 7:**
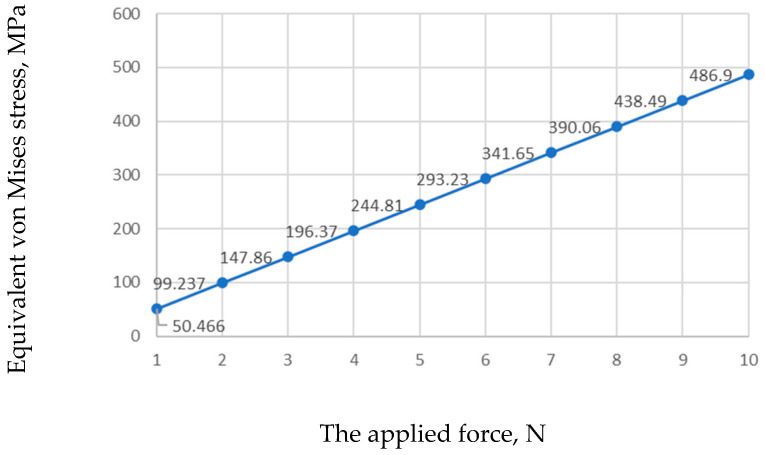
Maximum equivalent von Mises stress in the mini-implant.

In Figure 7, the graph shows the maximum equivalent von Mises stress in the global mini-implant model as a function of loading force. The maximum recorded value was 486.9 MPa in the most highly stressed and the minimum value was 50.466 MPa for a 1 N load. The yield strength of Ti-6Al-4V alloy varies significantly based on processing techniques and heat treatment conditions. For conventionally produced wrought Ti-6Al-4V, the average yield strength is approximately 800 MPa. However, additive manufacturing techniques can lead to a much higher yield strength, often exceeding 1000 MPa due to refined microstructures and residual stresses inherent in the process [28,29,30].

The graph indicates that the yield strength of the implant material is exceeded for forces above 9 N, suggesting that loads should remain below this threshold during orthodontic treatment. Considering that fatigue strength is typically lower than yield strength, the allowable load should be even smaller.

#### 3.2.2. Influence of Force Application Angle

##### Effect of Force Application Angle on Total Displacement

**Figure 8:**
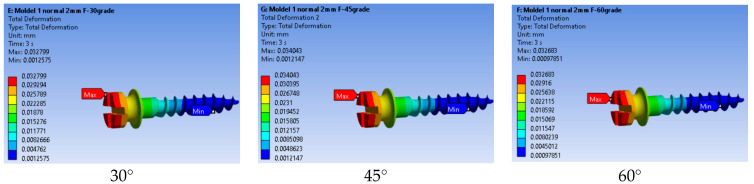
Total deformation of the mini-implant as a function of force application angle.

Figure 8 presents the total deformation of the mini-implant under three force application angles (30°, 45°, and 60°).

The total deformation decreased progressively as the force application angle increased from 30° to 60°. The maximum displacement was observed at 30° (0.032799 mm), while the smallest deformation occurred at 60° (0.032683 mm). This reduction in displacement can be attributed to a decrease in the bending moment acting at the implant neck as the loading direction becomes more vertical.

**Figure 9:**
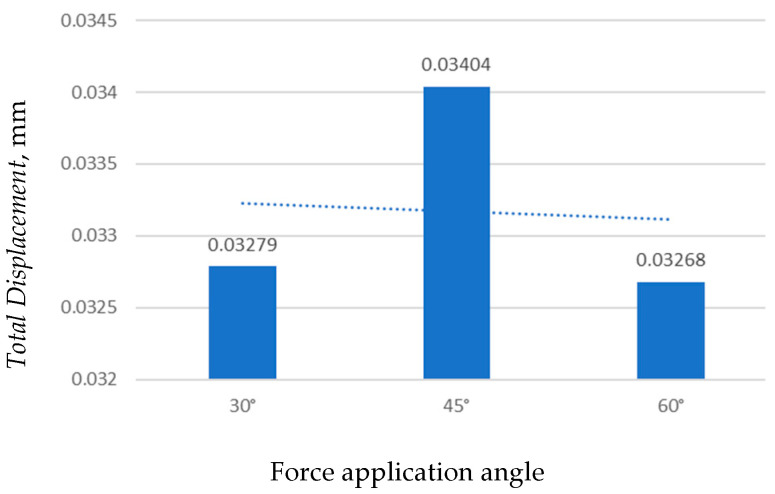
Maximum total deformation of the mini-implant vs. orthodontic force angle.

Figure 9 shows minor variations in maximum displacement depending on the force direction. The highest value was recorded at 45°, while 30° and 60° produced slightly lower responses. As the loading angle increases, a greater component of the force is transmitted along the implant axis, reducing bending stresses.

##### Equivalent Von Mises Stress

**Figure 10:**
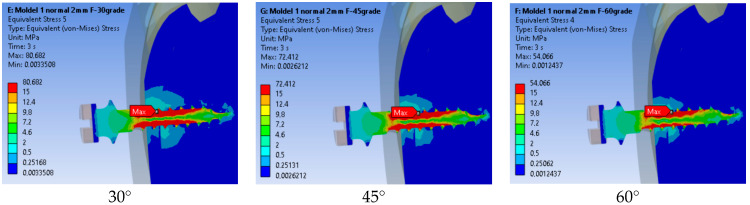
Stress distribution for three loading angles.

In Figure 10 the stress distribution for the three loading angles is presented. The maximum stress value (80.682 MPa) occurred at 30°, and the minimum (54.066 MPa) occurred at 60°. The stress distribution patterns were similar across all cases, with the maximum localized at the same point. However, at 60°, the stress values across both the bone and implant were lower.

**Figure 11:**
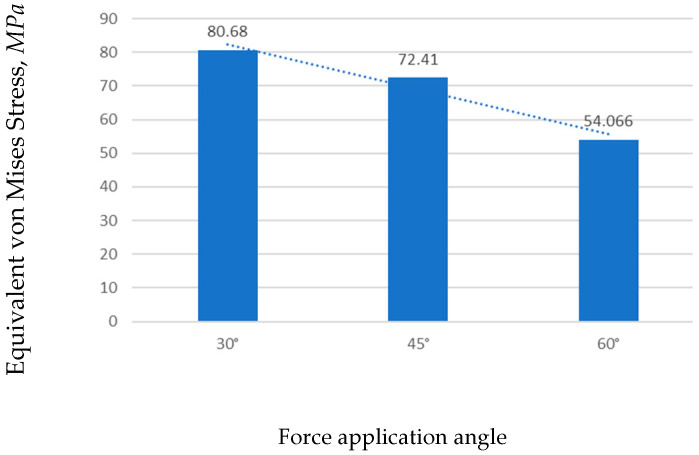
Comparison of maximum stress values by loading angle.

As illustrated in Figure 11, the stress in the mini-implant decreased as the force angle increased. The highest stress occurred at 30°, while at 60° the values were noticeably lower, indicating that higher loading angles help distribute the load more evenly and reduce concentration of stress near the implant neck.

##### Equivalent Strain

**Figure 12:**
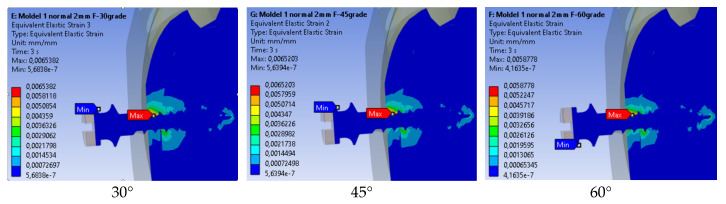
Equivalent linear strain for three loading angles.

Figure 12 shows the equivalent linear strain for the three loading angles. The maximum strain (0.0065382 mm/mm) was obtained for a 30° force, and the minimum (0.0058778 mm/mm) was obtained for 60°. As with stress, strain patterns were similar across models, with lower strain levels at higher loading angles.

**Figure 13:**
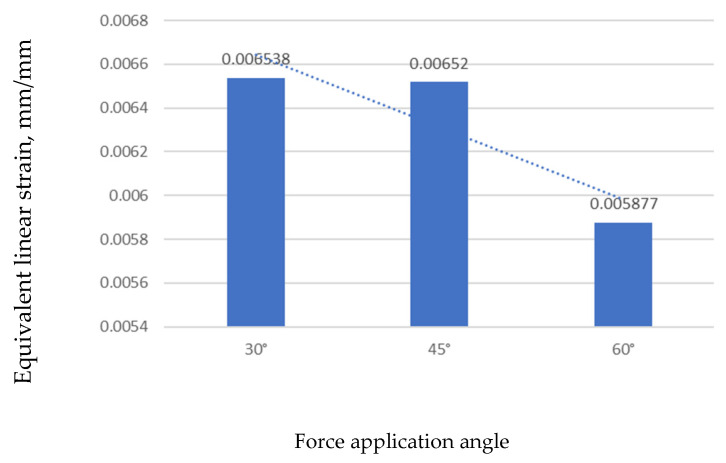
Comparison of maximum strain values by loading angle.

As shown in Figure 13, the strain values gradually decreased as the force angle increased. The highest strain appeared at 30°, and the lowest appeared at 60°, indicating that higher angles help distribute the load more evenly and reduce deformation around the mini-implant.

### 3.3. Influence of Mini-Implant Insertion Depth

#### 3.3.1. Total Displacement

As the mini-implant is inserted deeper, its movement under load becomes smaller, indicating greater stability. This trend emphasizes the importance of sufficient insertion depth for minimizing deformation and maintaining secure anchorage during orthodontic treatment.

**Figure 14:**
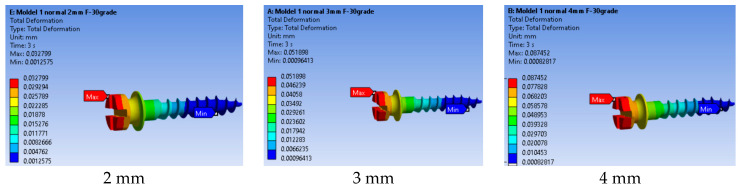
Total deformation in the mini-implant according to insertion depth.

Figure 14 shows the total deformation in the mini-implant depending on insertion depth (2 mm, 3 mm, and 4 mm). Larger deformations occur for shallower insertion depths, as the effective lever arm of the orthodontic force increases.

**Figure 15:**
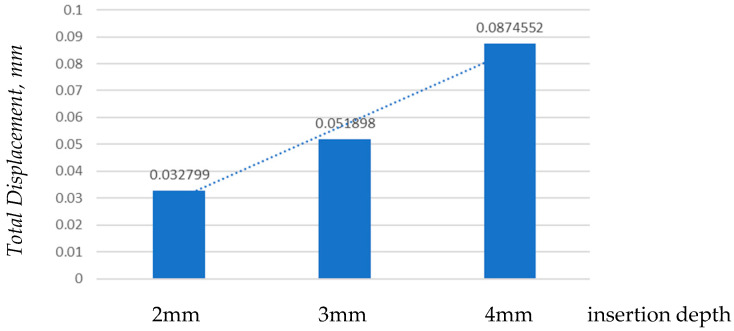
Comparison of maximum total displacements by insertion depth.

As shown in Figure 15, total displacement decreased as insertion depth increased. The highest movement occurred at 2 mm, while 4 mm provided the most stable result. This indicates that deeper insertion improves implant stability by reducing bending and the micromovement during orthodontic loading.

#### 3.3.2. Equivalent Von Mises Stress

**Figure 16:**
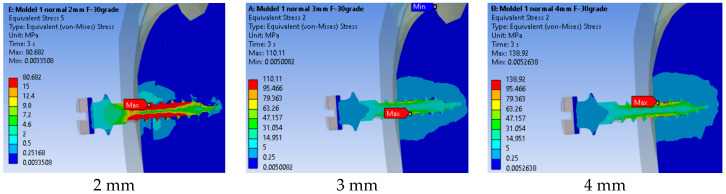
Stress distribution for three insertion depths.

Figure 16 shows the von Mises stress distribution for three insertion depths. The peak stress was localized in a very small region near the implant neck. When averaged over the adjacent volume, the stress distribution followed the same decreasing trend with increasing insertion depth. The stress patterns differ notably, with the maximum located closer to the surface for shallower insertion. A larger material volume is affected in both cortical and cancellous bone for shorter insertions.

**Figure 17:**
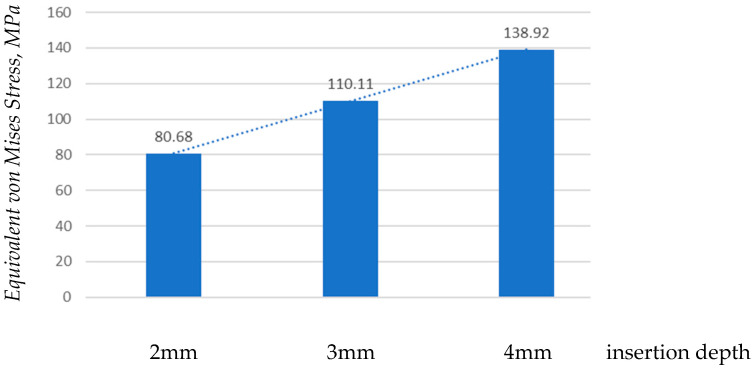
Comparison of maximum stress values by insertion depth.

As shown in Figure 17, the maximum stress increases progressively with insertion depth. The recorded values were 80.68 MPa at 2 mm, 110.11 MPa at 3 mm, and 138.92 MPa at 4 mm. This indicates a direct relationship between insertion depth and stress magnitude.

#### 3.3.3. Equivalent Strain

**Figure 18:**
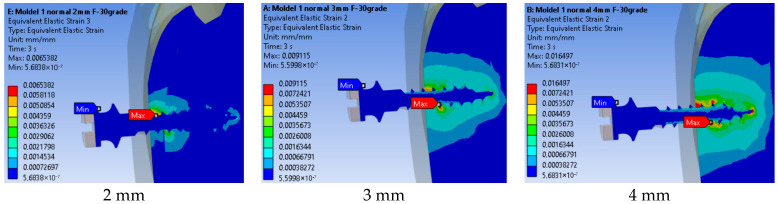
Equivalent linear strain for three insertion depths.

The distribution of equivalent linear strain for the three insertion depths is shown in Figure 18. The maximum value obtained was 0.016497 mm/mm for the insertion depth of 2 mm, while the minimum value was 0.0065382 mm/mm for the 4 mm insertion depth. The results indicate that the equivalent strain patterns differ among all analyzed cases, with the maximum values located at different points.

Table 4 summarizes the maximum displacement, von Mises stress, and equivalent strain values recorded for the different force angles and insertion depths analyzed in this study. The table allows direct comparison of biomechanical responses under var-ying loading configurations.

## 4. Discussion

### 4.1. Contact Pressure and Interfacial Load Transfer

Our model predicted a maximum contact pressure of 5.1 MPa along the thread–cortex interface (Figure 3). The concentration of contact pressure near the implant neck and upper threads suggests that these regions play a dominant role in load transfer under oblique orthodontic loading. This pattern is consistent with previous FEM studies reporting increased bending moments at the cervical region of mini-implants. Limited sliding areas indicate localized micromovements, which may influence primary stability under higher force magnitudes. This value sits at the lower bound of the ranges commonly reported for titanium miniscrews in cortical bone (≈20–40 MPa reported in several FEM/experimental studies under comparable loading). Two factors likely explain this difference: (i) the contact formulation (allowing separation with compression transfer) which disperses peak pressure over a broader thread flank and (ii) the force level actually used in clinical orthodontics (1–2 N in most scenarios).

In the context of orthodontic mini-implants, the maximum contact pressures reported for titanium implants in cortical bone typically range from about 19.85 MPa to 43.34 MPa, depending on specific dimensions and loading conditions. Sivamurthy and Sundari conducted a finite element analysis that revealed stress values for mini-implants with dimensions of 1.3 × 6 mm and 1.3 × 8 mm, showing a minimum stress of 19.85 MPa and a maximum of 43.34 MPa during retraction and intrusion, which are well within the fatigue limit of titanium, approximately 193 MPa [7] (Table 5). This indicates that mini-implants maintain structural integrity under typical orthodontic loads.

Zhou et al. corroborated these findings, noting that loading stress is primarily distributed in the cortical bone around the implant neck, with minimal stress in the bone around the implant root [31]. Their research highlighted this stress distribution, reinforcing the concept that it serves as a protective mechanism for the surrounding bone tissue. This is consistent with findings by Wahengbam et al., which reported that the concentration of forces in cortical bone is significant, and stresses are managed within the material limits of titanium [32].

Further detailing how the application of forces affects the interface, Yazıcıoğlu et al. found that oblique loads significantly enhance interfacial stresses [33]. Their findings revealed that oblique forces could elevate these stresses by as much as 5 to 20 times compared to vertical loads. They specifically analyzed the consequences of a 300 N oblique occlusal bite force on the mini-implant, confirming high localized stress at the facial and lingual areas of the bone, which is relevant for understanding potential points of failure. Lu et al. examined the impact of mini-implant characteristics—such as diameter and length—on stress levels in surrounding bone [25]. This suggests that optimizing implant dimensions may mitigate stress concentrations at the bone–implant interface. Sharma et al. explored the effects of insertion height and angulation on stress distribution at the mini-implant site [6].

The present FEM model predicted lower contact pressures (≈5.1 MPa) than previously reported ranges (≈20–40 MPa), mainly due to the nonlinear contact formulation and clinically relevant low orthodontic loads (1–2 N). Literature consensus indicates that stresses are concentrated around the implant neck and first threads, with magnitudes depending on implant geometry, insertion angle, and loading direction. This comparison reinforces that cortical bone is the dominant load-bearing region in mini-implant biomechanics.

### 4.2. Loading Direction

#### 4.2.1. Mechanism of Load Angle Influence

The progressive reduction in stress and equivalent strain with increasing loading angle can be explained by load vector decomposition. The applied force F can be resolved into an axial component Faxial = F cos θ and a transverse component F⊥ = F sin θ [34,35,36,37].

The transverse component governs bending and generates a bending moment at the implant neck, which can be approximated as M ≈ F ⊥ d = (F sin θ) d, where ddd is the effective lever arm. As θ increases, the bending-dominated component is reduced and the load becomes more axial, which decreases cervical bending stresses and promotes a more uniform transfer of load over the engaged threads, resulting in lower peak stress and strain.

#### 4.2.2. Comparison with Previous Studies on Loading

Across the 30°, 45°, and 60° loading directions—defined relative to a line parallel to the mini-implant axis in the sagittal plane—the finite element model revealed lower peak stress and strain values at 60°. Specifically, the maximum von Mises stress decreased from approximately 80.68 MPa at 30° to 54.07 MPa at 60°, while the equivalent strain also declined from 0.00654 to 0.00588 mm/mm. Although some studies have reported higher interfacial stresses under oblique (non-axial) loads, others have observed reduced stress at certain angled configurations. These discrepancies are likely due to differences in angle definition (relative to the implant axis, bone surface, or occlusal plane), load application points, and boundary or contact conditions. In the present model, a 60° loading direction minimized the bending moment at the implant neck, allowing a more uniform load distribution along the engaged threads and reducing stress concentration. Clinically, this suggests that the optimal force vector should minimize neck bending rather than adhere to a specific universal angle; under the current geometric and boundary conditions, a 60° direction achieved the most favorable stress distribution.

The optimal insertion angles for orthodontic mini-implants have been examined in various studies, with recommendations typically favoring angles between 30° and 45° for enhanced stability. Lin et al. reported that the suitable insertion angle range for mini-implants is between 30° and 45°, emphasizing that using these angles minimizes stress on the surrounding cortical bone while maximizing stability [38]. They conducted finite element analyses demonstrating that angles beyond this range, particularly 60°, tended to increase stress and, consequently, the risk of implant failure.

Popa et al. indicated that an insertion angle of 30° was beneficial, allowing better stress distribution compared to steeper angles like 60° or 90° [39]. Their research showed that the combination of insertion angle and cortical bone thickness significantly affects the stability of mini-implants, asserting that lower angles provide more favorable conditions for integration. However, Wilmes et al. concluded that traditional recommendations suggested avoiding low angles like 30° for mini-implants, and later findings indicate that angles between 60° and 70° may enhance primary stability while still maintaining low stress on the cortical bone [40]. This presents a contrast to the typical preference for 30° to 45° angles. Kovuru et al. found that altering the insertion angle from 90° to 45° increased cortical bone contact and improved primary stability for mini-implants [2]. Their findings support the preference for moderate angles (approximately 45°) to address osseointegration effectively. Xavier et al. discovered that incrementing the angle from 30° to 90° led to decreased stress values in both mini-implants and cortical bone, suggesting that higher angles could promote stability [41]. However, it is important to note that their study indicated an optimal angle closer to 90° for enhanced stability, which differs from the 30° to 45° recommendation. On the other hand, Marimuthu et al. presented evidence showing maximum stress in the bone at 30° and 60°, warning that excessive angling (up to 90°) could lead to increased stress and potential failure [42].

### 4.3. Insertion Depth

#### 4.3.1. Mechanism of Insertion Depth Influence

The reduction in displacement and equivalent strain with increasing insertion depth can be explained by changes in lever arm length and structural stiffness. A deeper insertion reduces the effective free length of the mini-implant above the cortical anchorage, thereby decreasing the bending moment (M = Fd) at the cervical region.

#### 4.3.2. Comparison with Previous Studies on Insertion Depth

Varying threaded engagement from 2 mm to 4 mm markedly altered system rigidity. At 2 mm, the maximum equivalent strain in bone reached 0.01650 mm/mm and the affected material volume was larger and closer to the cortical surface (Figure 12), with bone strain in cortex ≈2.5× higher than at 4 mm (Figure 13). These findings mirror reports that deeper insertion reduces lever-arm effects, spreads load over more threads, and shrinks the high-strain zone—all hallmarks of improved primary stability.

The literature provides various insights on optimal insertion depths for achieving primary stability for orthodontic mini-implants, with a consensus generally supporting deeper insertions as essential for enhanced stability. Pan et al. emphasized that mini-implants should be inserted as deeply as possible to maximize stability. They suggest that greater insertion depth not only increases stability but also minimizes tipping moments at the bone rim that could contribute to implant failure due to excessive strain at the bone–implant interface [43] (Table 6). Wilmes and Drescher discussed that higher insertion depths tend to yield greater insertion torques and primary stability; however, their focus was primarily on the effects of insertion angle and cortical bone thickness rather than explicitly on depth [40] (Table 6).

Ichinohe et al. confirmed that increasing insertion depth significantly enhances mini-implant stability. Their findings demonstrate that stability improves with depths greater than 6 mm, emphasizing the relation between depth and stability during the initial healing phase [45] (Table 6).

Hirai et al. indicated that an insertion depth of 4.1 mm could be optimal for maintaining adequate stability without increasing risk factors associated with screw fracture. This highlights a threshold where additional depth does not proportionately contribute to stability [46] (Table 6). However, the relationship with risk does not apply to all scenarios, as deeper insertions might still be required in specific cases. Nienkemper et al. underlined the importance of insertion depth, concluding that optimal depths should exceed 6 mm for effective primary stability, reinforcing the idea that deeper insertions correlate positively with retention and stability at the bone–implant interface [47] (Table 6).

### 4.4. Study Limitations

This finite element study is subject to certain limitations.

- The finite element model relied on simplified representations of bone geometry and material properties, which may not fully capture the structural complexity of human bone.
- This study did not include direct experimental validation. The model was verified by comparison with previously published finite element analyses rather than in vitro or in vivo measurements. Empirical validation is essential to assess the clinical applicability of finite element models. Previous studies have reported varying degrees of agreement between FEA predictions and experimental findings. For example, Mazhari et al. demonstrated a strong correlation between simulated stress distributions and experimental measurements when evaluating different miniscrew–tooth connection types, supporting the predictive capacity of FEA in orthodontic biomechanics [48].Conversely, Mešić et al., through experimental pull-out testing of mini-implants, showed that while FEA accurately reflected relative differences in implant behavior, quantitative discrepancies were observed depending on implant design and insertion technique [49]. These findings suggest that although finite element analysis is a valuable tool for comparative and parametric evaluation, its results should be interpreted with caution in the absence of direct experimental validation.
- Bone and implant materials were modeled as homogeneous and isotropic to allow numerical convergence, although this does not reflect their true anisotropic behavior. The assumption of isotropy in finite element models overlooks this intrinsic anisotropic nature, which could lead to inaccurate predictions of stress distributions and failure points. Studies indicate that explicitly including anisotropic material properties in finite element models enhances the prediction accuracy of local stress distributions significantly [50,51].
- Static loading conditions were applied, without simulating cyclic forces, fatigue behavior, or time-dependent biological processes such as bone remodeling. These factors may affect the long-term clinical performance of orthodontic mini-implants.
- The finite element model was based on a single mandibular geometry and therefore does not account for inter-individual anatomical variability. Differences in cortical bone thickness, trabecular density, and implant positioning may influence stress and strain distribution in clinical scenarios. Consequently, the present findings should be interpreted as representative of a specific anatomical configuration rather than universally applicable conditions.
- Biological processes, including bone remodeling and inter-individual anatomical variability, were not incorporated into the model.

## 5. Conclusions

Within the constraints of this finite element model, optimal mechanical performance was observed at moderate orthodontic forces (1–3 N), a loading direction of approximately 60°, and insertion depths of 2–4 mm. Under these simulated conditions, stress and strain levels in cortical and cancellous bone remained within physiological limits. These findings represent computational trends based on the specific geometric and material assumptions of the model and should be interpreted accordingly. Further experimental and in vivo studies are required to validate these results and confirm their clinical applicability.

## Figures and Tables

**Table 1:** The model of material properties used in the finite element analysis [21].

Material/Component	Young’s Modulus (MPa)	Poisson’s Ratio
Mini-implant (Ti-6Al-4V)	110,000	0.30
Cortical Bone	17,000	0.30
Cancellous Bone	350	0.25

**Table 2:** The main parameters used in the finite element analysis.

FEA Parameter	Property
Discretization (nodes/elements)	356,422/229,672
Element Type	10-node tetrahedral element
Software	ANSYS
Material Model	Isotropic, homogeneous, and linear elastic
Contact Model	Frictional (nonlinear, friction coefficient = 0.3 µ between mini-implant and bone); bonded (linear)
Loading	Oblique (0.1–10 N) (30°, 45°, 60°)

**Table 3:** Number of elements and nodes (FEA simulation—real clinical case).

Model Component	Elements	Nodes
Mini-implant	2644	5184
Bracket	1556	3007
Teeth	53,879	81,478
PDL	3681	7383
Cortical bone	57,004	95,034
Cancellous bone	110,721	163,906
Adhesive	187	430
Total	229,672	356,422

**Table 4:** Summary of peak stress, strain, and displacement values under different loading conditions.

Parameter	Scenario	Max Displacement (mm)	Max von Mises Stress (MPa)	Max Equivalent Strain (mm/mm)
Force angle	30°	0.0328	80.682	0.0065382
	45°	0.03404	—	—
	60°	0.0267	54.066	0.0058778
Insertion depth	2 mm	Highest displacement	—	0.016497
	3 mm	—	—	—
	4 mm	Lowest displacement	80.682	0.0065382

**Table 5:** Summary of contact pressure and interfacial load transfer findings: present FEM results versus previous studies.

Present FEM Study (2025)	Findings from Literature
Max contact pressure: 5.1 MPa	Typical range: 19.85–43.34 MPa
Contact localized along thread–cortex interface	Sivamurthy & Sundari (2016) [7]: 1.3 × 6 mm and 1.3 × 8 mm implants; stresses within titanium fatigue limit (~193 MPa).
Load mainly in cortical region near implant neck	Zhou et al. (2018) [31]: stress concentrated at neck; minimal stress near root.
Pressure values lower than literature range	Wahengbam et al. (2022) [32]: force concentration in cortical bone; within safe material limits.
- Nonlinear contact with separation	Yazıcıoğlu et al. (2015) [33]: oblique loads increase stress by 5–20×; high local stresses at facial/lingual bone.
- Lower orthodontic forces (1–2 N)	Lu et al. (2015) [25]: larger diameter leads to lower cortical stress.

**Table 6:** Insertion Depth and Stability.

Parameter/Finding	Present Fem Study (2025)	Findings from Literature	References
Insertion depth analyzed	2 mm–4 mm	Typically 4–7 mm in most studies	Pan et al. [43] Petrey et al. [44]
Maximum equivalent strain	0.01650 mm/mm at 2 mm; ~2.5× higher than at 4 mm	Higher strain at shallower insertions due to increased lever arm effect	Ichinohe et al. [45];
Implant displacement	Increased at 2 mm; minimal at 4 mm	Deeper insertion reduces micromovement and bending	Petrey et al. [44] Hirai et al. [46]
Stress distribution pattern	Larger strained volume near cortical surface at 4 mm; reduced at 2 mm	Deeper engagement distributes load across more threads and reduces cortical stress	Pan et al. [43]; Wilmes & Drescher [40]
Predicted stability	2–4 mm insertion depth provides best rigidity and lowest strain	Depths ≥ 6 mm generally enhance torque and pull-out resistance	Nienkemper et al. [47]; Ichinohe et al. [45]

## Data Availability

The original contributions presented in this study are included in the article. Further inquiries can be directed to the corresponding author.

## Abbreviations

The following abbreviations are used in this manuscript:

FEM	Finite Element Method
FEA	Finite Element Analysis
MI	Mini-implant
CT	Computed Tomography
CAD	Computer-Aided Design
Ti-6Al-4V	Titanium alloy (90% Ti, 6% Al, 4% V)
PDL	Periodontal Ligament
von Mises stress	Equivalent stress criterion (no abbreviation, but often used as term)
MPa	Megapascal
mm	Millimeter
N	Newton
STL	Standard Tessellation Language (file format for 3D printing/geometry)
ANSYS	Analysis System (commercial FEM software)
3D	Three-dimensional
°	Degrees (angle measurement)
